# circHIPK3 regulates lung fibroblast-to-myofibroblast transition by functioning as a competing endogenous RNA

**DOI:** 10.1038/s41419-019-1430-7

**Published:** 2019-02-22

**Authors:** Jia-xiang Zhang, Jian Lu, Hui Xie, Da-peng Wang, Huan-er Ni, Yong Zhu, Le-hao Ren, Xiao-xiao Meng, Rui-lan Wang

**Affiliations:** 1 Department of Critical Care Medicine, Shanghai General Hospital, Shanghai Jiaotong University, School of Medicine, Shanghai, China; 2 Department of Critical Care Medicine, Shanghai General Hospital of Nanjing Medical University, Shanghai, China; 3 Department of Cardiology, Shanghai General Hospital, Shanghai Jiaotong University, School of Medicine, Shanghai, China

## Abstract

Myofibroblasts predominantly emerging through fibroblast-to-myofibroblast transition (FMT) are considered to be the key collagen-producing cells in pulmonary fibrosis. Circular RNAs (circRNAs) are important players involved in many biological processes. circHIPK3 has been identified as the one of the most abundant circRNAs in human lung. In this study, we characterized the role of circHIPK3 in pulmonary fibrosis. We revealed that circHIPK3 is upregulated in bleomycin-induced pulmonary fibrosis mice model, FMT-derived myofibroblasts. circHIPK3 silencing can ameliorate FMT and suppress fibroblast proliferation in vivo and vitro. Fundamentally, circHIPK3 regulates FMT by functioning as an endogenous miR-338-3p sponge and inhibit miR-338-3p activity, thereby leading to increased SOX4 and COL1A1 expression. Moreover, dysregulated circHIPK3 expression was detected in the clinical samples of patients with idiopathic pulmonary fibrosis. Intervention of circHIPK3 may represent a promising therapy for pulmonary fibrosis.

## Introduction

Idiopathic pulmonary fibrosis (IPF) is a chronic, progressive, and lethal disease characterized by the aberrant accumulation of fibrotic tissue in the lung parenchyma^1^. Although the disease has been considered rare, the incidence of IPF is similar to that of stomach, brain, and testicular cancers. The median survival time from diagnosis is only 2–4 years, which is shorter than various types of cancers^2^. The pathogenesis of IPF is incompletely understood and current therapies are limited to those that reduce the rate of functional decline in partial patients^3^. Therefore, novel agents targeted to halt the fibrotic process are urgently needed.

Formation of fibrotic foci that consist of myofibroblasts and the aberrant expression of extracellular matrix (ECM) proteins in the lungs is a prominent pathologic characteristic of IPF. Studies have demonstrated that the accumulation of myofibroblasts is predominantly from resident tissue fibroblasts^4–7^. Thus, understanding the regulatory mechanism of fibroblast-to-myofibroblast transition (FMT) process in IPF would provide novel therapeutic targets for IPF.

Circular RNAs (circRNAs) are novel class of non-coding RNAs that are covalently closed continuous loops. The majority of circRNAs are highly abundant, stable, and conserved across species, and often exhibit tissue-specific expression pattern^8^. In recent studies, functional circRNAs have been shown to participate in the regulatory networks governing gene expression at transcriptional and post-transcriptional level. Aberrant circRNA expressions have been implicated in several human diseases, especially those proliferative diseases such as tumorigenesis^9^. Inspired by these findings, we speculate circRNAs are potential regulators of IPF. Manipulation of circRNAs may open up a novel avenue for molecular therapeutics of IPF.

circHIPK3 was verified as one of the most abundant circRNAs in each human tissues^10^. Aberrant circHIPK3 expression has been implicated in many solid tumors^11–14^. Moreover, previous study has reported that circHIPK3 is enriched in human fibroblast^15^. However, whether circHIPK3 participates in mediating the biological function of fibroblast remains elusive.

In this study, by utilizing a bleomycin (BLM)-induced IPF experimental model, we characterized the expression pattern of circHIPK3 and investigated for the first time its role in fibroblast proliferation and pulmonary FMT. We revealed that silencing circHIPK3 could resulted in inhibition of fibroblast proliferation and differentiation into myofibroblasts in vivo and in vitro. Intervention of circular RNA would provide novel insights into the therapeutics of IPF.

## Methods and materials

### Cell culture and treatments

WI-38 cells and HEK-293T were purchased from American Type Culture Collection (Rockville, MD, USA). WI-38 and Cells HEK-293T were cultured in Dulbecco’s modified Eagle’s medium containing 10% fetal bovine serum (Gibco) at 37 ℃ in 5% CO_2_ and 95% humidity. For FMT assay, WI-38 cells were treated with 10 ng/ml human recombinant TGF-β1 (PeproTech, Rocky Hill, NJ, USA) for 48 h with or without designated agents^16^.

### BLM-induced pulmonary fibrosis model

Eight- to ten-week old male C57BL/6 J mice were purchased from the Chinese Academy of Sciences experiment centre in Shanghai. Animals were housed in the Laboratory Animal Center of Shanghai General Hospital (Shanghai, China). All of the animal studies were approved by the Shanghai Jiaotong University Animal Care and Use Committee. For BLM-induced fibrosis studies, mice under anesthesia were administered a single intratracheal injection of BLM sulfate (3 mg/kg; Selleckchem, http://www.selleckchem.com). Control mice were given sham treatment with saline. Mice were harvested 4 weeks after BLM treatment. Parts of lung lobes were fixed in 4% paraformaldehyde for histopathologic analyses, parts were frozen for subsequent immunoblotting and immunofluorescent studies. Lung microsections (5 μm) were stained with Masson’s trichrome and Sirius red to visualize fibrotic lesions.

### circHIPK3 shRNA adeno-associated virus (AAV) production and intratracheal injection

Three different shRNAs were designed for circHIPK3 silencing by Vigene Biosciences, Inc. The sequences of shRNAs as shown in Supplementary Table 1. shRNA3 with best silence efficiency was chosen for animal experiment. For AAV production, shRNA3 or scrambled sequences were inserted into AAV vector. C57BL/6 J mice (5-week-old, male) under anesthesia were intratracheally administered about 30 μL (2.34 × 10^13^ viral particles/ml) AAV6 containing circHIPK3 shRNA or scrambled shRNA. After 3 weeks, mice were challenged with BLM for pulmonary fibrosis experiments.

### Immunofluorescence experiment

WI-38 cells or lung microsections (5 μm) were fixed in 4% paraformaldehyde, permeabilized with 0.1% Triton X-100 for 10 min at room temperature, and blocked in 5% bovine serum albumin for 60 min. The cells or lung microsections were incubated with primary antibody at 4 ℃ overnight, and then incubated with secondary antibody for 1 h at room temperature after three washes with TBST. Nuclei were stained with DAPI for 3 min. Finally, the cells or lung microsections were observed under a laser confocal microscope (Leica TCS SP8; Leica, Wetzlar, Germany). Images from each sections were measured in a blinded manner and quantified using Image-Pro Plus software (Media Cybernetics, Silver Spring, MD). The following primary antibodies and dilutions were used for immunofluorescence microscopy experiments: α-SMA (1:50, ab7817; Abcam), desmin (1:50, ab15200; Abcam), Ki67 (1:100, 556003; BD Biosciences). The following secondary antibodies were used: anti-mouse-Alexa Fluor 647 (1:1000, Invitrogen), anti-rabbit-Alexa Fluor 488(1:1000, Invitrogen).

### RNase R treatment and RT-PCR

Two microgram of total RNA was incubated 20 min with or without 3 U/μg of RNase R (Epicentre), and the resulting RNA was extracted using Trizol reagent (Invitrogen, Carlsbad, CA). circHIPK3 was validated by semiquantitative reverse transcription polymerase chain reaction (RT-PCR) using the PrimeScript™ RT-PCR kit (Takara, Dalian, China).

### Real-time quantitative PCR

Total RNAs were extracted from tissues and cells using Trizol reagent (Invitrogen, Carlsbad, CA). The concentration of total RNA was detected using an ultraviolet spectrophotometer. Total RNA samples were reversely transcribed to cDNAs using the reverse transcription kit (Takara, Dalian, China) according to the manufacturer’s protocol. Real-time quantitative PCRs were conducted using SYBR Green PCR kit (Toyobo, Osaka, Japan) and ABI ViiA™ 7 System. The expression levels of target gene were normalized to GAPDH expression levels by using 2^-ΔΔCT^ method. The specific divergent primers for circHIPK3 and other primers used in our study are shown in Supplementary Table 1.

### Western blotting

To detect the protein of interest, the cell-derived lysates and lung-derived protein samples were separated through 10% SDS-PAGE electrophoresis and transferred to PVDF membranes. The blots were then blocked with 5% non-fat milk powder in TBST buffer for 1 h, and incubated at 4 ℃ overnight with the appropriate primary antibodies at appropriate dilutions. After washing, the bolts were incubated with HRP-conjugated secondary antibody for 1 h at room temperature. The bands were visualized using the ECL system (Bio-Rad). The used antibody included α-SMA (1:1000, ab7817; Abcam), collagen 1α1 (1:1000, 84336 S; CST).

### Oligonucleotide transfection

siRNAs were synthesized by GenePharma (Shanghai, China). miRNA mimics were synthesized by Ribobio (Guangzhou, China). The sequences used are show in Supplementary Table 1. The cells transfections were conducted using Lipofectamine RNAiMax (Thermo Fisher Scientific). WI-38 cells were transfected with 50 nM siRNAs or miRNA mimics for 48 h, and then treated with 10 ng/ml TGF-β1 for 48 h.

### Cell viability assay

Cell proliferation was tested using EdU (5-ethynyl-2’-deoxyuridine) DNA Cell Proliferation Kit (RiboBio, Guangzhou, China) and CCK8 kit (Doindo, Japan). After required treatment, WI-38 cells were incubated with 50 nM EdU for another 2 h. The proliferating cells were fixed with 4% paraformaldehyde and incubated with Apollo Dye Solution. Cell nuclei were stained with Hoechst 33342. For CCK8 assay, transfected cells were plated in 96-well plates at a density of 5 × 10^3^ cells per well. After required treatment, CCK8 reagent was added to each well to incubate for another 2 h. The absorbance was measured at 450 nm wavelength using a microplate reader (Thermo Fisher, NewYork, USA).

### circRNA plasmids construction and transfection

Human circHIPK3 cDNA was synthesized and cloned into pADM-circRNA-GFP vector (Vigene Biosciences, Shandong, China). The pADM-circRNA-GFP vector contained a front circular frame and a back circular frame. Transfection was carried out using Lipofectamine RNAiMax (Thermo Fisher Scientific) according to the manufacturer’s instructions.

### RNA fluorescence in situ hybridization

FITC-labelled RNA probes to circHIPK3, Cy3-labelled miR-338-3p were designed and synthesized by GenePharma (Shanghai, China), and the probes sequences were shown in Supplementary Table 1. Signals were detected by Fluorescent in situ Hybridization Kit (RiboBio, Guangzhou, China) according to the manufacturer’s instructions. The images were acquired under a laser confocal microscope (Leica TCS SP8; Leica, Wetzlar, Germany).

### RIP

The RIP assay was conducted using the Magna RIP™ RNA-Binding Protein Immunoprecipitation Kit (Millipore, Bedford, MA). RIP was performed using WI-38 cell lysate and either anti-Ago2 (2897 T, CST) or Normal Rabbit IgG as the immunoprecipitating antibody. Purified RNA was then analyzed by qRT-PCR or/and RT-PCR by using specific primers.

### Luciferase reporter assay

The 3’-UTR or mutant 3’-UTR of circHIPK3, SOX4 and COL1A1 were separately inserted into the downstream of the luciferase gene in the pmirGLO-control vector (Vigene Biosciences, Shandong, China). HEK-293T cells were plated at 5 × 10^3^ per well in 96-well plates. The cells were co-transfected with Firefly, Renilla luciferases, circHIPK3 plasmids, miRNA mimics, and negative control mimics at appropriate concentration. The luciferase activity was detected using a dual luciferase reporter assay system (Promega, Madison, WI). For comparison, the Firefly luciferase activity was normalized to Renilla luciferase activity.

### Statistical analysis

The data are presented as the means ± SEM or means ± SD. The comparisons between any two groups were performed using a two-tailed unpaired *t*-test for normally distributed data. Multiple group comparisons were performed using one-way ANOVA with the Tukey post test to determine the differences among all groups. The significance level was set at *P* < 0.05.

## Results

### circHIPK3 is upregulated in murine model of pulmonary fibrosis

circbase retrieval shows that one of isoform of circHIPK3 is highly conserved between human and mouse genome, which is located at chr11:33307958–33309057 in human genome (hsa_circ_0000284) and chr2:104310905–104312004 in mouse genome (mmu_circ_0001052). We focus on this isoform for further study.

**Fig. 1:**
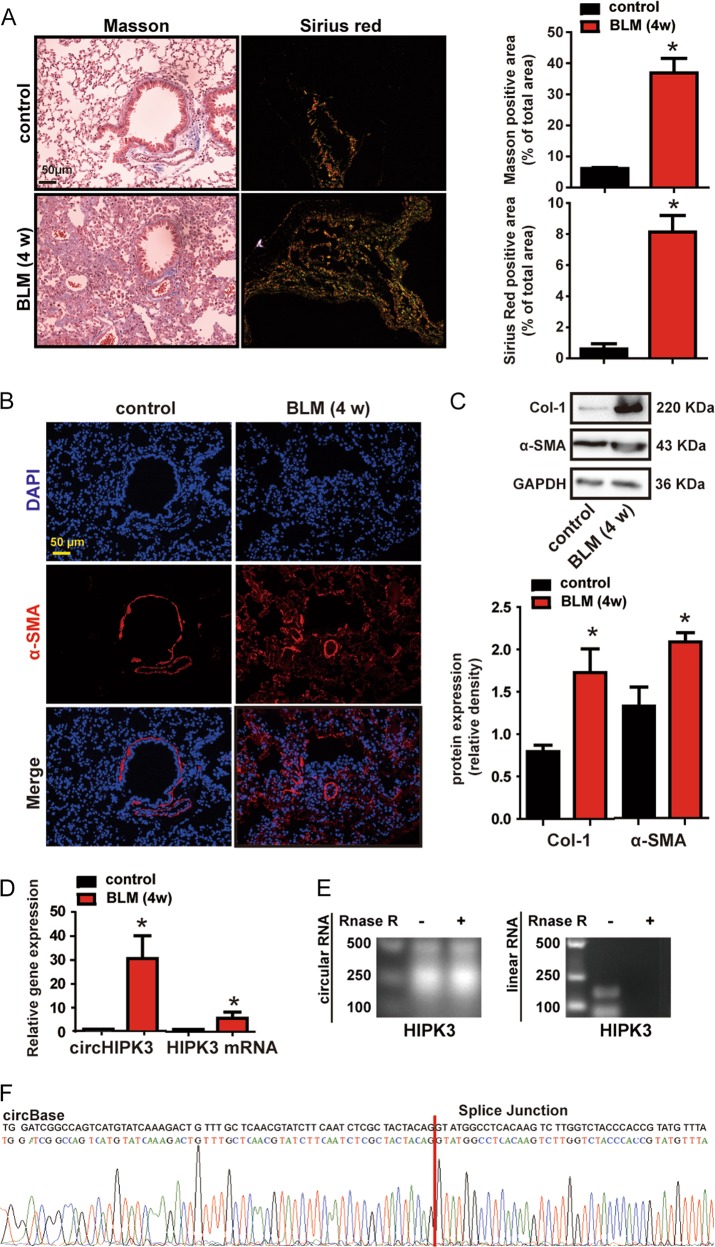
circHIPK3 is upregulated in murine model of pulmonary fibrosis. **a** Lung tissue sections from normal and bleomycin-treated mice were stained with Masson’s trichrome or Sirius red. Quantification of positive staining area was performed (*n* = 4, *p < 0.05 vs. control). Scale bar, 50 μm. **b** Lung tissue sections from normal and bleomycin (BLM)-treated mice were stained with myofibroblast marker α-SMA. Red, α-SMA; blue, nuclei. Scale bar, 50 μm. **c** Myofibroblast markers, Col-1 and α-SMA in mouse lung were determined by western bolt. GAPDH was detected as the internal control. A representative immunoblot was shown (*n* = 4, *p < 0.05 vs. control). **d** qRT-PCR was performed to detect the expression of circHIPK3 and HIPK3 mRNA in the lung of normal and bleomycin-treated mice (*n* = 4, *p < 0.05 vs. control). **e** Total RNAs isolated from normal mouse lung were digested by RNase R followed by RT-PCR detection of mouse circHIPK3 and HIPK3 mRNA. **f** The sequence of mouse circHIPK3 was obtained using Sanger sequencing. Data are represented as means ± SD

We established pulmonary fibrosis mouse model with a single intratracheal administration of bleomycin (BLM). Lungs were examined 28 days after injury. BLM caused severe pulmonary fibrosis and collagen deposition (Fig. 1a), and as expected, BLM caused increased myofibroblast accumulation, since we detected increased markers of myofibroblast including alpha smooth muscle actin (α-SMA) and Type I Collagen (Col-1) (Fig. 1b, c). We further evaluated the circHIPK3 expression pattern in the lung lysates. Quantitative RT-PCR (qRT-PCR) showed that circHIPK3 was significantly higher in the lung of BLM-induced pulmonary fibrosis mice, compared with that of control mice (Fig. 1d). The HIPK3 mRNA expression also exhibited significant upregulation, however, the change fold was much smaller than circHIPK3 (Fig. 1d). We assessed the stability of circHIPK3 through RNase R treatment. circHIPK3 (mmu_circ_0001052) was resistant to RNase R digestion, while linear HIPK3 mRNA was easy to degrade (Fig. 1e). The RT-PCR product was sent for sanger sequencing. The sequencing result was completely consistent with circHIPK3 (mmu_circ_0001052) in circBase (Fig. 1f). Besides circHIPK3, circSLC8A1, circGSE1, and circBMPR2 are also abundant in human lung and show high homology between human and mouse genome^11^. Mouse circSLC8A1, circGSE1, and circBMPR2 are resistance to the digestion of RNase R exonuclease compared to their linear isoform, which confirmed that these RNAs are circular in form (Supplementary Fig 2, A). We then evaluated the expression of circSLC8A1, circGSE1, and circBMPR2 in the lung of BLM-induced pulmonary fibrosis mice by qRT-PCR. However, no significant difference was observed in the expression of circSLC8A1, circGSE1, and circBMPR2 in the fibrosis model, which indicates the absence of a general induction of circRNAs (Supplementary Fig 2, B). Collectively, these findings indicate a strong correlation between circHIPK3 and pulmonary fibrosis.

### circHIPK3 is upregulated in FMT-derived myofibroblasts

**Fig. 2:**
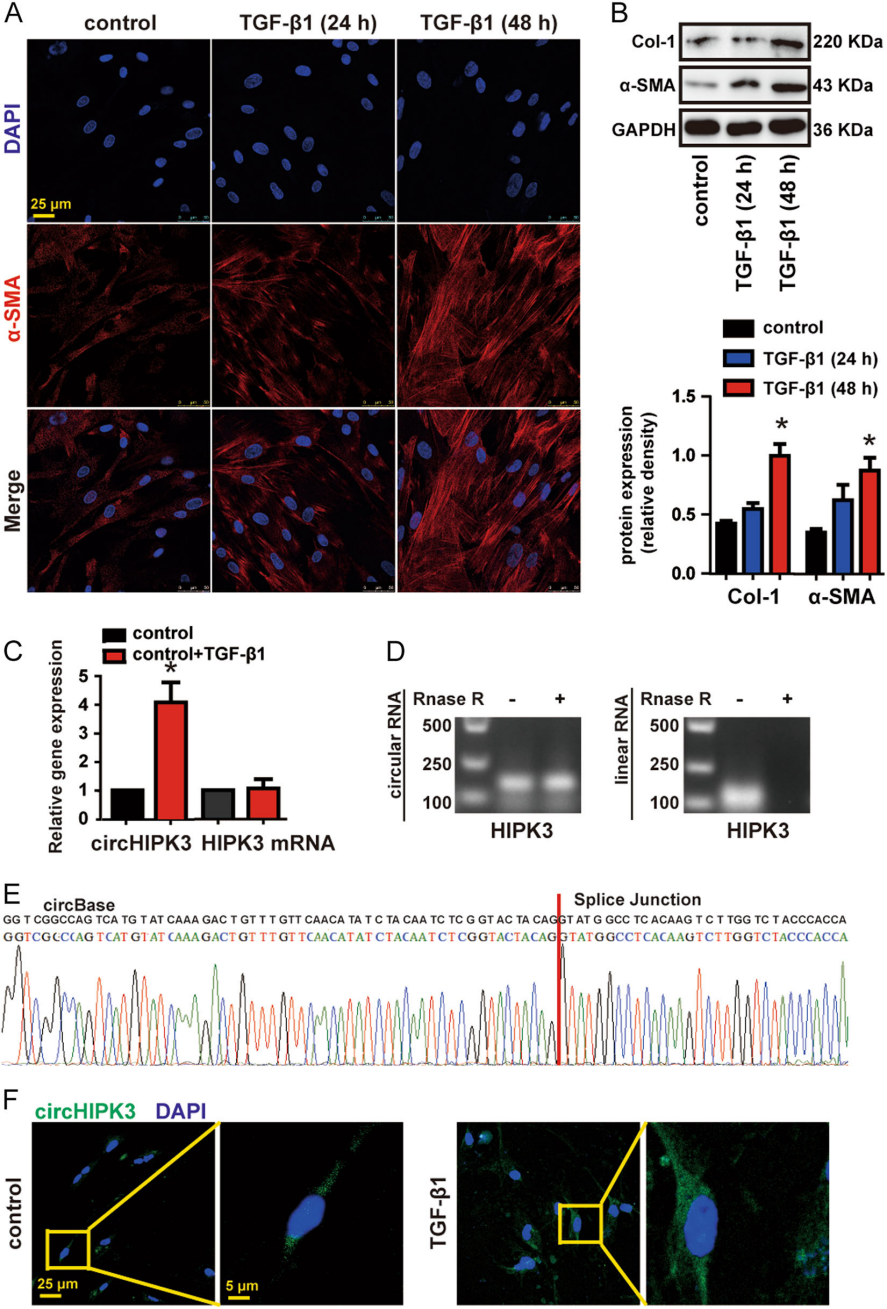
circHIPK3 is upregulated in FMT-derived myofibroblasts. **a** WI-38 cells were treated with 10 ng/ml TGF-β1 for 24 or 48 h or left untreated (control). Immunofluorescence assays were performed to detect the myofibroblast marker α-SMA. Red, α-SMA; blue, nuclei. Scale bar, 25 μm. **b** Myofibroblast markers, Col-1 and α-SMA were determined by western bolt. GAPDH was detected as the internal control. A representative immunoblot was shown (*n* = 3, *p < 0.05 vs. control). **c** WI-38 cells were treated with 10 ng/ml TGF-β1 for 48 h or left untreated (control). qRT-PCR was performed to detect the expression of circHIPK3 and HIPK3 mRNA (*n* = 3, *p < 0.05 vs. control). **d** Total RNAs isolated from WI-38 cells were digested by RNase R followed by RT-PCR detection of human circHIPK3 and HIPK3 mRNA. **e** The sequence of human circHIPK3 was obtained using Sanger sequencing. **f** RNA-FISH assay was conducted to detect the expression distribution of circHIPK3 in WI-38 cells. Green, circHIPK3; blue, nuclei. Scale bar, left:25 μm; right: 5 μm. Data are represented as means ± SEM

FMT-derived myofibroblasts are the major source of ECM proteins during pathological matrix remodeling in pulmonary fibrosis^5^. Importantly, persistent transforming growth factor-β (TGF-β) signaling is the primary mechanism of FMT and fibrosis^17^. We then investigated circHIPK3 expression pattern during TGF-β1-induced FMT process. In our experimental condition, WI-38 cells treatment with 10 ng/ml TGF-β1 for 48 h markedly increased myofibroblast marks α-SMA and Col-1 (Fig. 2a, b). qRT-PCR analysis indicated that circHIPK3 was significantly upregulated in TGF-β1-treated fibroblast compared to vehicle alone, whereas linear HIPK3 mRNA was not significantly altered (Fig. 2c). By combining RT-PCR analysis that used divergent primers targeting the circular junction and sanger sequencing, we confirm the circHIPK3 derived from WI-38 is in accordance with circHIPK3 (hsa_circ_0000284) in circBase (Fig. 2e). circHIPK3 (hsa_circ_0000284) was resistant to RNase R digestion, whereas the liner isoform was not (Fig. 2d). Fluorescence in situ hybridization (FISH) assays revealed increase fluorescence intensity of circHIPK3 (hsa_circ_0000284) staining in TGF-β1-treated fibroblast compared to vehicle alone (Fig. 2f). Notably, circHIPK3 (hsa_circ_0000284) was mainly expressed in the cytoplasm of WI-38 (Fig. 2f). These findings show that circHIPK3 is upregulated in FMT-derived myofibroblasts.

### circHIPK3 silencing attenuates BLM-induced pulmonary fibrosis and ameliorates fibroblast dysfunction in vivo

**Fig. 3:**
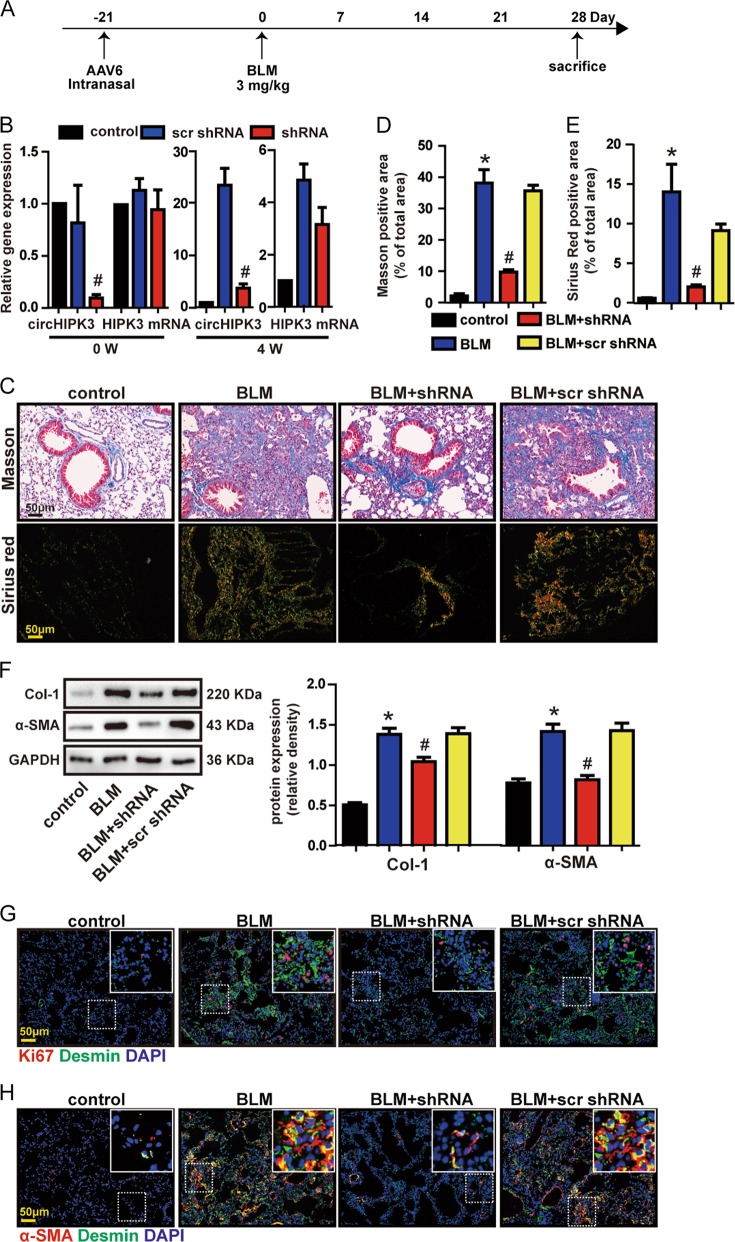
circHIPK3 silencing attenuates BLM-induced pulmonary fibrosis and ameliorates fibroblast dysfunction in vivo. **a** Strategy for knockdown circHIPK3 in bleomycin (BLM)-induced pulmonary fibrosis mouse model. Five-week old male mice were received an intratracheal injection of circHIPK3 shRNA, scrambled shRNA (scr shRNA) or left untreated. Three weeks later, mice were received a single intratracheal administration of BLM or saline. **b** The expressions of circHIPK3 and HIPK3 mRNA in mice lung at the indicated time points were detected by qRT-PCR (*n* = 4, ^#^*p* < 0.05 vs. scr shRNA). **c** Lung fibrosis was determined by Masson’s trichrome or Sirius red staining. Scale bar, 50 μm. **d,** **e** Quantification of positive staining area was measured and statistically analyzed (*n* = 4, *p < 0.05 vs. control, ^#^*p* < 0.05 vs. scr shRNA). **f** Myofibroblast markers, Col-1 and α-SMA were determined by western bolt. GAPDH was detected as the internal control. A representative immunoblot was shown (*n* = 4, *p < 0.05 vs. control, ^#^*p* < 0.05 vs. scr shRNA). **g** Immunofluorescence analysis of Ki67 and desmin were conduct to assess the lung fibroblast proliferation. Red, Ki67; Green, desmin; blue, nuclei. Scale bar, 50 μm; insets, 12.5 μm. **h** Immunofluorescence analysis of α-SMA and desmin were performed to assess the lung fibroblast-to-myofibroblast differentiation. Red, α-SMA; Green, desmin; blue, nuclei. Scale bar, 50 μm; insets, 12.5 μm. Data are represented as means ± SD

To reveal the role of circHIPK3 in pulmonary fibrosis, we designed three adeno-associated viral shRNAs for circHIPK3 silencing. All of them could obviously reduce circHIPK3 expression levels (Supplementary Fig 1, A). We chose shRNA3 for further in vivo experiment due to its silencing efficiency (Supplementary Fig S1, A and B). circHIPK3 shRNAs intratracheal injection significantly reduced lung circHIPK3 but not liner HIPK3 mRNA expression throughout the experiment (Fig. 3b). circHIPK3 silencing could attenuate collagen deposition in BLM-induced pulmonary fibrosis mice model, as demonstrated by Masson’s trichrome and Sirius red staining (Fig. 3c–e). In accordance, α-SMA and Col-1 expression were significantly decreased in circHIPK3 knockdown mice after bleomycin injury (Fig. 3f). These results suggest that circHIPK3 silencing ameliorates BLM-induced pulmonary fibrosis and ECM deposition.

Fibroblasts in IPF or mice models are heterogeneous, suggesting different celluar origins or different stages of activation^18^. Desmin is an intermediate filament protein, which can mark stromal cells with a fibroblastic morphology. We observed increased desmin-positive fibroblast cells in fibrotic mice lungs (Fig. 3g and Supplementary Fig 1, C). Ki67 staining showed increased proliferation of desmin-positive fibroblast cells in fibrotic mice lungs (Fig. 3g and Supplementary Fig S1, D). These results indicate that lung cells including fibroblasts proliferate in response to bleomycin injury. Whereas, circHIPK3 silencing reduced the profibrotic effect of bleomycin as shown by decreased total Ki67-positive cells in lung (Fig. 3g and Supplementary Fig 1, E). To be specific, the amount of proliferation cell nuclear antigen (Ki67) was lower in desmin-positive fibroblast cells, which demonstrated that circHIPK3 silencing could suppress the proliferation of fibroblast in fibrotic lung (Fig. 3g and Supplementary Fig 1, D). Moreover, bleomycin also caused increased desmin-positive fibroblast cells gained myofibroblast marker (α-SMA), which verified the involvement of FMT in fibrotic process (Fig. 3h and Supplementary Fig 1, F). Notably, inhibition circHIPK3 significantly prevented bleomycin-induced FMT process (Fig. 3h and Supplementary Fig 1, F). Taken together, circHIPK3 knockdown alleviates fibroblast proliferation and FMT in vivo.

### circHIPK3 regulates FMT and fibroblast proliferation in vitro

**Fig. 4:**
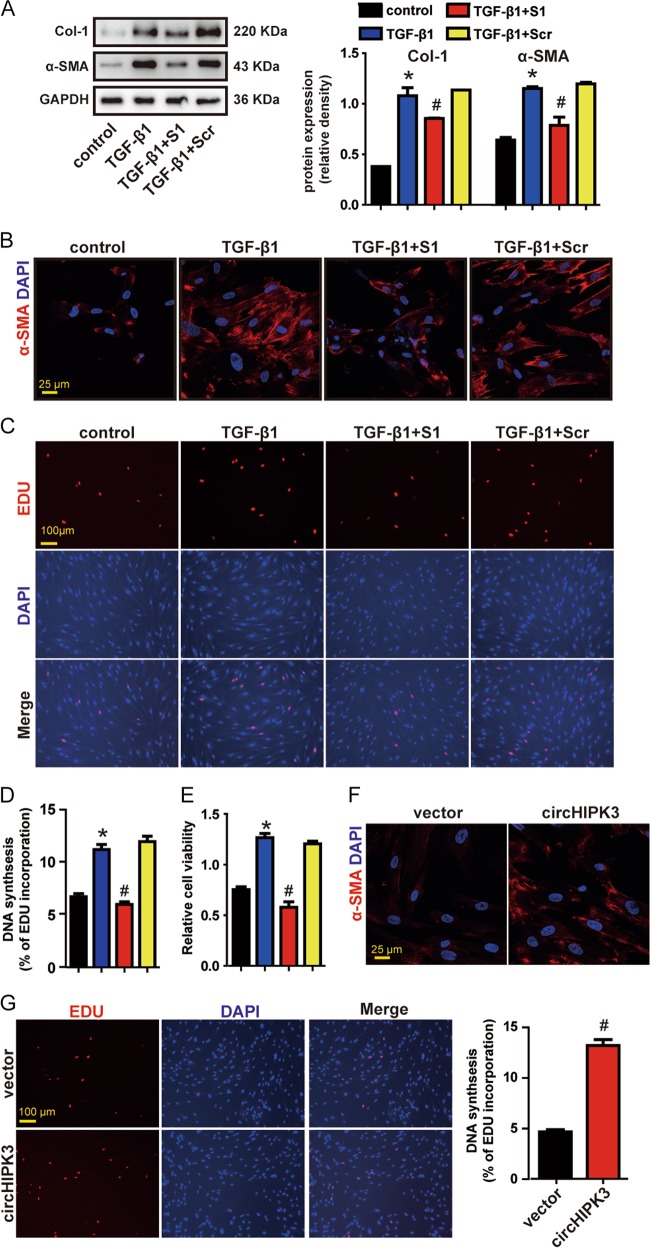
circHIPK3 regulates FMT and fibroblast proliferation in vitro. **a** WI-38 cells were treated with circHIPK3 siRNA1 (S1), scrambled siRNA (Scr) or left untreated and then incubated with 10 ng/ml TGF-β1 for 48 h. Myofibroblast markers, Col-1 and α-SMA were determined by western bolt. GAPDH was detected as the internal control. A representative immunoblot was shown (*n* = 3, *p < 0.05 vs. control, ^#^*p* < 0.05 vs. Scr). **b** Immunofluorescence assays were performed to detect the myofibroblast marker α-SMA. Red, α-SMA; blue, nuclei. Scale bar, 25 μm. **c** DNA synthesis was assessed using EDU assay. Red, EDU; blue, nuclei. Scale bar, 100 μm. **d** EDU assay data were quantified (*n* = 3, *p < 0.05 vs. control, ^#^*p* < 0.05 vs. Scr). **e** Cell proliferation was assessed using CCK8 assay (*n* = 3, *p < 0.05 vs. control, ^#^*p* < 0.05 vs. Scr). **f** WI-38 cells were transfected with pADM-GFP (vector) or pADM-circHIPK3-GFP. Immunofluorescence assays were performed to detect the myofibroblast marker α-SMA. Red, α-SMA; blue, nuclei. Scale bar, 25 μm. **g** DNA synthesis was assessed using EDU assay. Red, EDU; blue, nuclei. Scale bar, 100 μm. Data are represented as means ± SEM

TGF-β1 is the most potent profibrotic cytokine known to mediate the process of pulmonary fibrosis by accumulation of fibroblast and FMT^17^. We thus investigated if circHIPK3 is required for the activation of TGF-β1-induced FMT and TGF-β1-induced fibroblast proliferation. We designed two small interfering RNAs (siRNAs) targeting the backsplice sequence of circHIPK3. siRNA1 transfection significantly downregulated circHIPK3 expression without interfering the expression of linear HIPK3 mRNA (Supplementary Fig 1, G and H). We found that circHIPK3 silencing attenuated TGF-β1-induced expression of α-SMA and Col-1 (Fig. 4a, b). EDU incorporation assays revealed that knockdown of circHIPK3 significantly reduced TGF-β1-induced fibroblast proliferation (Fig. 4c, d). CCK8 assays revealed that circHIPK3 silencing significantly decreased the viability of WI-38 cells under TGF-β1 treatment (Fig. 4e). We conclude that circHIPK3 is essential for the activation of TGF-β1-induced FMT and TGF-β1-induced fibroblast proliferation.

We then examined whether circHIPK3 is sufficient alone to regulate fibroblast functions by conducting gain-of-function experiments. Overexpression of circHIPK3 upregulated the expression of α-SMA and Col-1 (Fig. 4f and Supplementary Fig 1, J). EDU incorporation assays revealed that overexpression of circHIPK3 significantly increased the proliferation of WI-38 cells (Fig. 4g). These findings indicate that overexpression of circHIPK3 is sufficient to induce FMT and aggravate fibroblast proliferation.

### circHIPK3 regulates FMT and fibroblast proliferation by acting as a miR-338-3p sponge

**Fig. 5:**
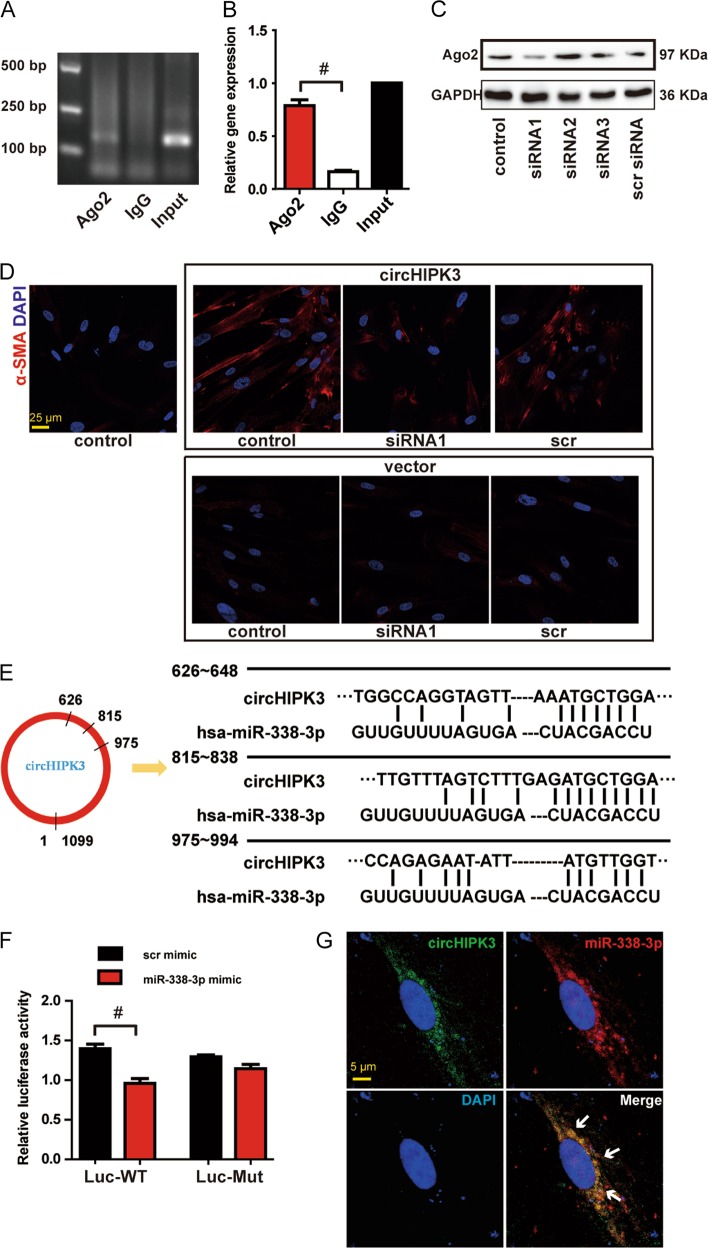
circHIPK3 regulates FMT by acting as a miR-338-3p sponge. **a** The total cell fractions were isolated from WI-38 cells and immunoprecipitated using Ago2 or IgG antibody. The expression of circHIPK3 was detected by RT-PCR. **b** The amount of circHIPK3 in the immunoprecipitate was measured by qRT-PCR (*n* = 3, ^#^*p* < 0.05 vs. IgG). **c** WI-38 cells were transfected with Ago2 siRNA1 (siRNA1), Ago2 siRNA2, Ago2 siRNA3, scrambled siRNA (scr) or left untreated. Western bolt was conducted to detect Ago2 expression. GAPDH was detected as the internal control. **d** WI-38 cells were transfected with Ago2 siRNA1, scrambled siRNA (scr) or left untreated plus pADM-GFP (vector) or pADM-circHIPK3-GFP. Immunofluorescence assays were performed to detect the myofibroblast marker α-SMA. Red, α-SMA; blue, nuclei. Scale bar, 25 μm. **e** Schematic of putative binding sites of miR-338-3p on circHIPK3 transcript. **f** HEK-293T were co-transfected with LUC-circHIPK3 or LUC-circHIPK3 mutant with miR-338-3p mimics or scrambled mimics. Luciferase activity was detected 48 h after transfection (*n* = 3, ^#^*p* < 0.05 vs. scr mimic). **g** RNA-FISH assay was conducted to detect the expression distribution of circHIPK3 and miR-338-3p in TGF-β1-treated WI-38 cells. Red, circHIPK3; Green, miR-338-3p; blue, nuclei. Scale bar, 5 μm. Data are represented as means ± SEM

circRNAs participate in mediating biological process by functioning as cytoplasmic miRNA sponges^8^. circHIPK3 has been reported to act as platform for Ago2 and miRNAs in HEK-293T^10^. To address whether circHIPK3 could sponge miRNAs in WI-38 cells, we performed RIP assays. RIP assays showed that circHIPK3 was enriched in Ago2-containing immunoprecipitates compared with control immunoglobulin G (IgG) immunoprecipitates (Fig. 5a, b), which confirmed the interaction between circHIPK3 and Ago2 in WI-38 cells. Ago2 is a core component of RNA-induced silencing complex (RISC), which can bind miRNA to target mRNA transcripts^19^. The RIP assays indicated that circHIPK3 in WI-38 could regulate FMT through sponging miRNAs. We then further investigated whether circHIPK3 regulated FMT in an Ago2-dependent manner. Three siRNAs were synthesized to knockdown Ago2, and siRNA1 with greater silencing efficiency was chosen for subsequent experiments (Fig. 5c). We found that circHIPK3 overexpression-induced FMT was interrupted when Ago2 was knockdown (Fig. 5d). These results suggest that circHIPK3 regulates FMT in an Ago2-dependent manner.

We then identified the miRNAs that binded to circHIPK3. Bioinformatics prediction using circNet database and Circular RNA Interactome database shows that miR-338-3p is a common miRNA that potentially binds to circHIPK3 (Fig. 5e). circHIPK3 cDNA was cloned into the downstream of luciferase gene (LUC-circHIPK3) and transfected into HEK-293T with miRNA mimics. The activity of LUC-circHIPK3 was significantly reduced by miR-338-3p, but not by scrambled mimic (Fig. 5f). miR-338-3p transfection had no effect on the activity of LUC-circHIPK3 mutant (Fig. 5f). FISH assays revealed that circHIPK3 and miR-338-3p were colocalized in the cytoplasm of TGF-β1-treated WI-38 cells (Fig. 5g). These results suggest that circHIPK3 serves as a binding platform for Ago2 and miR-338-3p, and may function as a miRNA sponge.

### circHIPK3-miR338-3p/SOX4/COL1A1 network is involed in regulating FMT

**Fig. 6:**
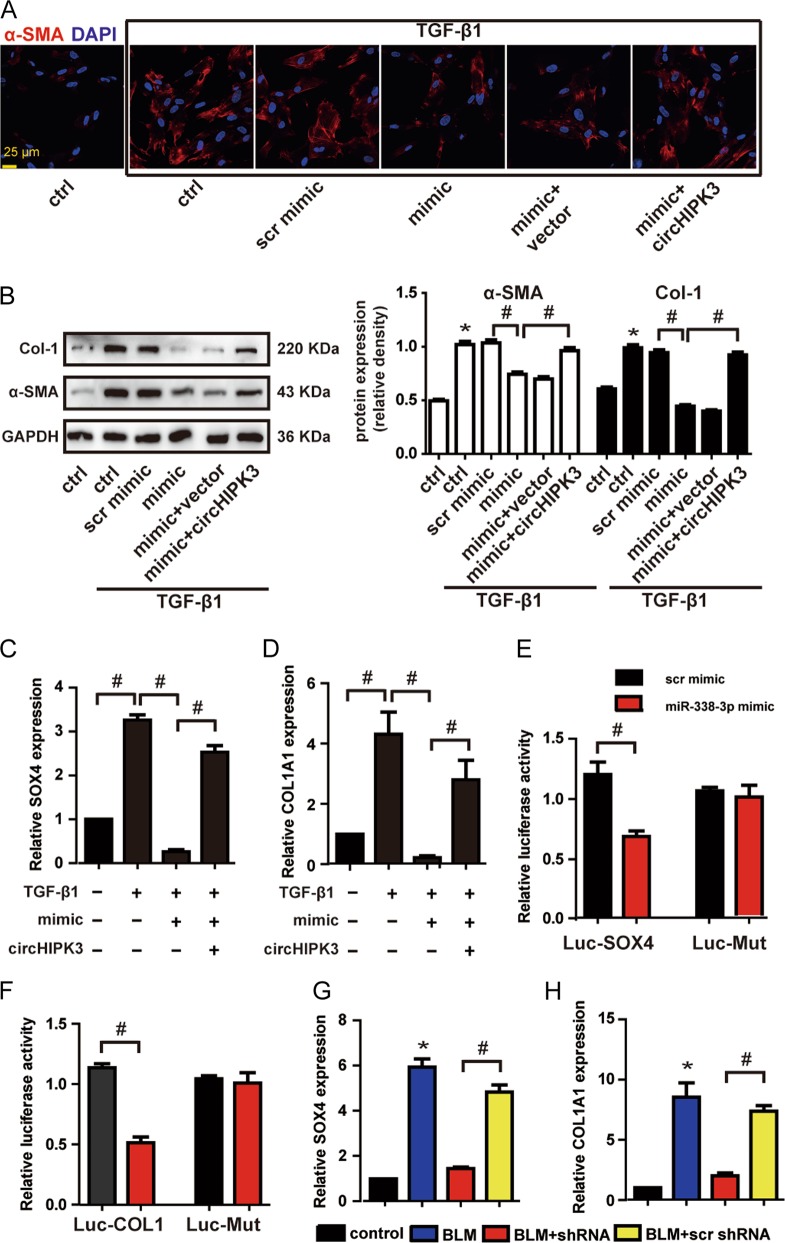
circHIPK3-miR338-3p/SOX4/COL1A1 network is involved in regulating FMT. **a** WI-38 cells were transfected with miR-338-3p mimics or scrambled mimics or left untreated plus pADM-GFP (vector) or pADM-circHIPK3-GFP, and then treated with TGF-β1 for 48 h. Immunofluorescence assays were performed to detect the myofibroblast marker α-SMA. Red, α-SMA; blue, nuclei. Scale bar, 25 μm. **b** Myofibroblast markers, Col-1 and α-SMA were determined by western bolt. GAPDH was detected as the internal control. A representative immunoblot was shown (*n* = 3, *p < 0.05 vs. control, ^#^*p* < 0.05 between the marked groups). **c,** **d** WI-38 cells were transfected with miR-338-3p mimics or left untreated plus pADM-circHIPK3-GFP, and then treated with TGF-β1 for 48 h. qRT-PCR was performed to detect the expression of SOX4 and COL1A1(*n* = 3, ^#^*p* < 0.05 between the marked groups). **e,** **f** HEK-293T were co-transfected with LUC-SOX4, LUC-COL1A1 or LUC-mutant with miR-338-3p mimics or scrambled mimics. Luciferase activity was detected 48 h after transfection (*n* = 3, ^#^*p* < 0.05 vs. scr mimic). **g,** **h**) The expression of SOX4 and COL1A1 in mice lung were detected by qRT-PCR (*n* = 4, *p < 0.05 vs. control, #p < 0.05 vs. scr shRNA). Data (**b**–**f**) are represented as means ± SEM. Data (**g,** **h**) are represented as means ± SD

Since miR-338-3p was sponged by circHIPK3, we then determined the role of miR-338-3p in vitro. miR-338-3p transfection could repress TGF-β1-induced protein expression of α-SMA and Col-1, whereas the repression was abrogated by circHIPK3 overexpression (Fig. 6a, b). These results indicate circHIPK3/miR-338-3p interaction is involved in regulating TGF-β1-induced FMT. We then employed Targetscan database to predict the target gene of miR-338-3p. Two candidate genes including SOX4 and COL1A1 were indentified. SOX4 has been confirmed as a master regulator of mesenchymal features^20,21^. Col-1 is the main components of ECM accumulated in fibrotic foci^22,23^. SOX4 and COL1A1 expression was significantly upregulated during TGF-β1-induced FMT process (Fig. 6c, d). miR-338-3p mimic transfection in the presence of TGF-β1 significantly inhibited SOX4 and COL1A1 expression (Fig. 6c, d). Moreover, the miR-338-3p-mediated repression of the two target genes was rescued by the circHIPK3 overexpression (Fig. 6c, d). The direct regulation of miR-338-3p on its target site was confirmed by luciferase report assay. Sequences of SOX4 and COL1A1 were, respectively, cloned into the downstream of luciferase gene (LUC-SOX4 and LUC- COL1A1) and transfected into HEK-293T with miR-338-3p mimic. The activity of LUC-SOX4 and LUC-COL1A1 was significantly reduced by miR-338-3p, but not by scrambled mimic (Fig. 6e, f). In the lung of BLM-induced pulmonary fibrosis mice, SOX4 and COL1A1 expression was significantly upregulated (Fig. 6g, h). In BLM-induced pulmonary fibrosis mice, circHIPK3 silencing significantly decreased SOX4 and COL1A1 expression (Fig. 6g, h). Collectively, these results reveal that circHIPK3-miR338-3p/SOX4/COL1A1 network is involved in regulating FMT.

### Clinical relevance of circHIPK3 in idiopathic pulmonary fibrosis

**Fig. 7:**
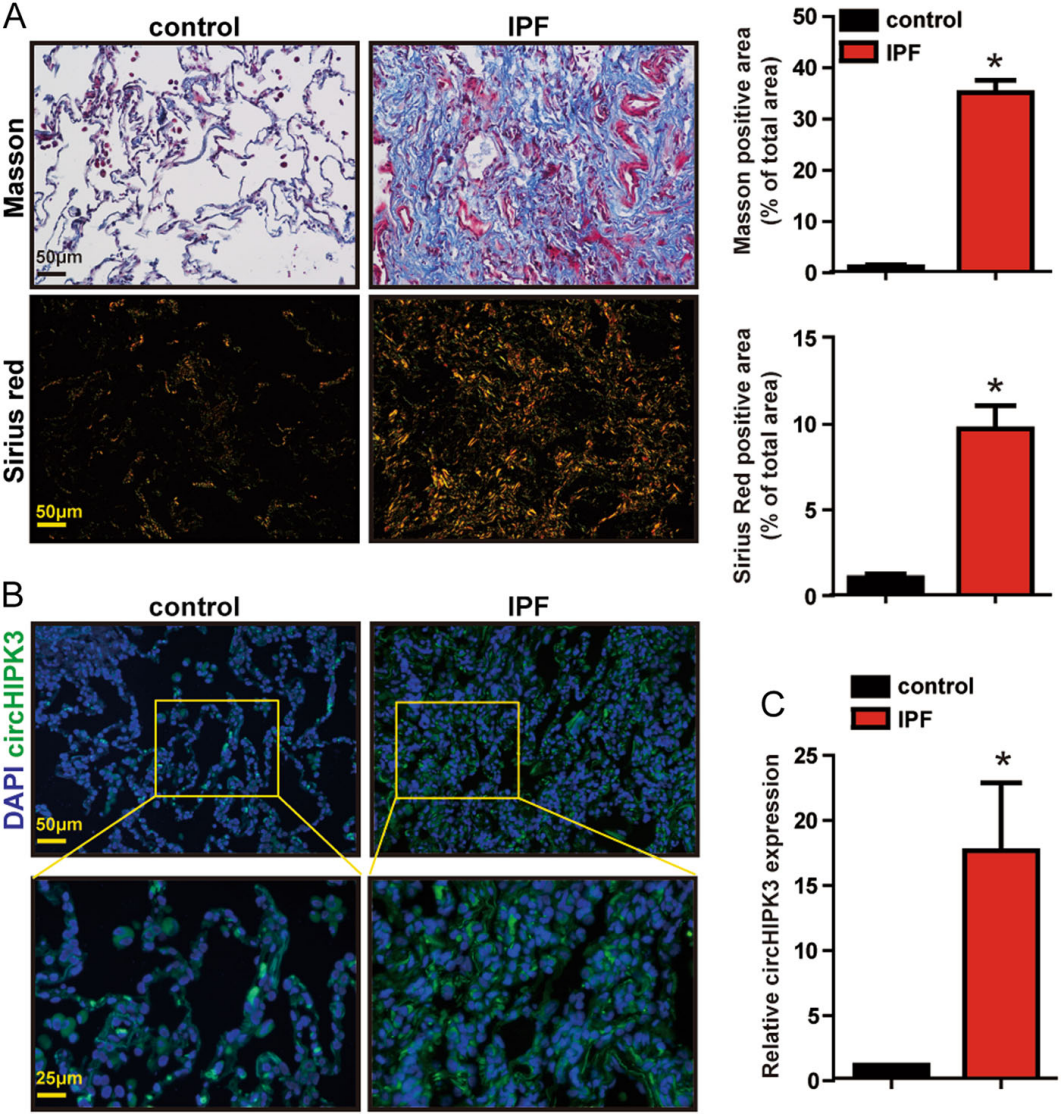
Clinical relevance of circHIPK3 in idiopathic pulmonary fibrosis. **a** Lung tissue sections from normal people and IPF patients were stained with Masson’s trichrome or Sirius red. Quantification of positive staining area was performed (*n* = 3, *p < 0.05 vs. control). Scale bar, 50 μm. **b** RNA-FISH assay was conducted to detect the expression distribution of circHIPK3 in lung tissues of normal people and IPF patients. Green, circHIPK3; blue, nuclei. Scale bar, upper: 50 μm; lower: 25 μm. **c** The expression of circHIPK3 in the lung tissues of normal people and IPF patients were detected by qRT-PCR. (*n* = 3, *p < 0.05 vs. control). Data are represented as means ± SD

We further investigated circHIPK3 dysregulation occurs in IPF patient lung tissue samples. Compared with control group, IPF patient lung tissue samples exhibited increased collagen deposition as shown by Masson’s trichrome and Sirius red staining (Fig. 7a–c). qRT-PCR and FISH assay revealed significantly upregulated circHIPK3 expression in IPF patient lung tissue samples (Fig. 7d, e). Collectively, these results indicate that circHIPK3 is potentially involved in the pathogenesis of IPF.

## Discussion

In this study, we identify that circHIPK3 is upregulated in BLM-induced pulmonary fibrosis mice model, FMT-derived myofibroblasts and idiopathic pulmonary fibrosis patient lung samples. We show that circHIPK3 silencing can ameliorate FMT and suppress fibroblast proliferation in vivo and vitro. We then present substantial evidence supporting that circHIPK3 can function as an endogenous miR-338-3p sponge and inhibit miR-338-3p activity, thereby leading to increased SOX4 and COL1A1 expression.

Emerging evidence has confirmed the relevance of circRNA to diverse types of human disease, such as tumorigenesis, neurological disorders, and cardiovascular diseases^24–27^. Previous studies have showed that circHIPK3 is one of the most abundant circRNAs in human lung^10^. However, the role of circHIPK3 in idiopathic pulmonary fibrosis remains elusive. We then extended circRNA study into a novel research field, fibrotic diseases. We first examine the expression pattern of circHIPK3 in fibrotic mouse lungs, FMT-derived myofibroblasts and idiopathic pulmonary fibrosis patient lung samples and investigate its functional role in pulmonary fibrosis.

Myofibroblasts are the dominant collagen-producing cells in pulmonary fibrosis. It has been reported that myofibroblasts could origin from multiple cellular sources such as alveolar epithelial cells via epithelial-to-mesenchymal transition (EMT), endothelial cells via endothelial-to-mesenchymal transition (EnMT), resident fibroblasts via fibroblast-to-myofibroblast transition (FMT), pericytes via activation, and circulating fibrocytes via recruitment^5^. Of these, resident fibroblasts are the major contributors to myofibroblasts in the lung^7^. Consistent with these findings, we observe that fibrotic mouse lungs and idiopathic pulmonary fibrosis patient lung samples exhibit increased myofibroblast markers α-SMA and Col-1, and this pathological change is accompanied by increased circHIPK3 expression. Notably, silencing circHIPK3 markedly decreases collagen deposition in vivo. More importantly, silencing circHIPK3 attenuates FMT and inhibits fibroblast proliferation in BLM-induced pulmonary fibrosis mouse model.

TGF-β1 is probably the most potent fibrogenic factor in pulmonary fibrosis. TGF-β1 drives the production of α-SMA, which endows myofibrobalast with contractility. However, direct TGF-β-based antifibrotic therapy has adverse immune effect^28–30^. Therefore, intervening the key downstream effectors of TGF-β pathway provides a therapeutic target for IPF. In this study, we discover that the treatment of TGF-β1 induce FMT in WI-38 cells. And the treatment of TGF-β1 significantly upregulates circHIPK3 but not its linear isoform in vitro. This result is supported by the notion that the more an exon is circularized, the less it presents in the processed mRNA^31–33^. Intriguingly, both circHIPK3 and HIPK3 mRNA expression are significantly increased in fibrotic mouse lungs. We thus speculate that additional regulators could affect exon circularization or skipping in linear isoforms in vivo condition.

Previous studies have revealed that circHIPK3 silencing could inhibit tumor cell proliferation^11,13,14^. We delineate similar role of circHIPK3 in WI-38 cells. In our study, through gain- and loss-of-function assays, we validate circHIPK3 was essential and sufficient for TGF-β1-induced fibroblast proliferation in vitro. Additionally, our results demonstrate circHIPK3 is a key regulator in TGF-β1-induced FMT in vitro. Contrary to our and other’s results, Yawei Li et al. reported that overexpression of circHIPK3 suppressed bladder cancer cells growth. We speculate circHIPK3 may have specific molecular function in each tissue type.

Our vivo and vitro evidences demonstrate that silencing circHIPK3 can reduce the development of pulmonary fibrosis through attenuate FMT and inhibit fibroblast proliferation. Thus, inhibition of circHIPK3 may represent a promising therapeutic approach for pulmonary fibrosis. We then further investigated the molecular mechanism by which circHIPK3 regulated FMT. circHIPK3 has been reported to act as a miRNA sponge to regulate gene expression. Consistent with previous studies, we confirm the ability of circHIPK3 to bind to Ago2, which indicates circHIPK3 can sequester miRNAs in WI-38 cells. However, other than acting as miRNA sponges, emerging biological functions of circRNAs have been recognized including binding to RNA-associated proteins and encoding functional proteins. We first question whether circHIPK3 perform its functions in an Ago2-depend manner. We discover that circHIPK3 overexpression-induced FMT is interrupted when Ago2 is knockdown. This result suggests circHIPK3 regulates FMT by acting as miRNA sponge.

Through bioinformatics analysis we identify miR-338-3p is a common miRNA that potentially binds to circHIPK3. miR-338-3p is highly conserved cross species. miR-338-3p has been identified as a tumor suppressor in several types of tumors^34–36^. In our study, we first determine the capacity of miR-338-3p to repress TGF-β1-induced FMT in WI-38 cells. We then provide several lines of evidence to prove the interaction between circHIPK3 and miR-338-3p. First, the luciferase activity of LUC-circHIPK3 can be significantly reduced by miR-338-3p. Second, our RNA-FISH assays show high abundance of circHIPK3 and miR-338-3p are overlapped in the cytoplasm of WI-38 cells. Finally, rescue assays demonstrate circHIPK3/miR-338-3p is critical for TGF-β1-induced FMT in WI-38 cells. miRNAs govern gene expression via degrading the target mRNA. We investigated two potential target genes of miR-338-3p. SOX4 has been reported as a key regulator of mesenchymal features^20,21^. COL1A1 encoding Col-1 is the main components of ECM accumulated in fibrotic foci^22,23^. Our results show upregulated SOX4 and COL1A1 expression in fibrotic mouse lungs, FMT-derived myofibroblasts. We reveal that circHIPK3-miR338-3p/SOX4/COL1A1 network is involved in regulating FMT. These observations strengthen our notion that circHIPK3 intervention may represent a promising target for pulmonary fibrosis.

## Conclusions

In conclusion, our study demonstrates circHIPK3 is upregulated in BLM-induced pulmonary fibrosis mice model, FMT-derived myofibroblasts, and idiopathic pulmonary fibrosis patient lung samples. circHIPK3 silencing can ameliorate FMT and suppress fibroblast proliferation in vivo and vitro. circHIPK3 regulates FMT by functioning as an endogenous miR-338-3p sponge and inhibit miR-338-3p activity, thereby leading to increased SOX4 and COL1A1 expression.

## Supplementary information

Supplementary Figure 1

Supplementary Figure 2

supplemental figure legends

Supplementary Table 1
