# Supplementary figures and images for: circHIPK3 regulates lung fibroblast-to-myofibroblast transition by functioning as a competing endogenous RNA
Source: Cell Death Dis. 2019 Feb 22;10(3):182. doi: 10.1038/s41419-019-1430-7 (PMC6385182; doi:10.1038/s41419-019-1430-7)

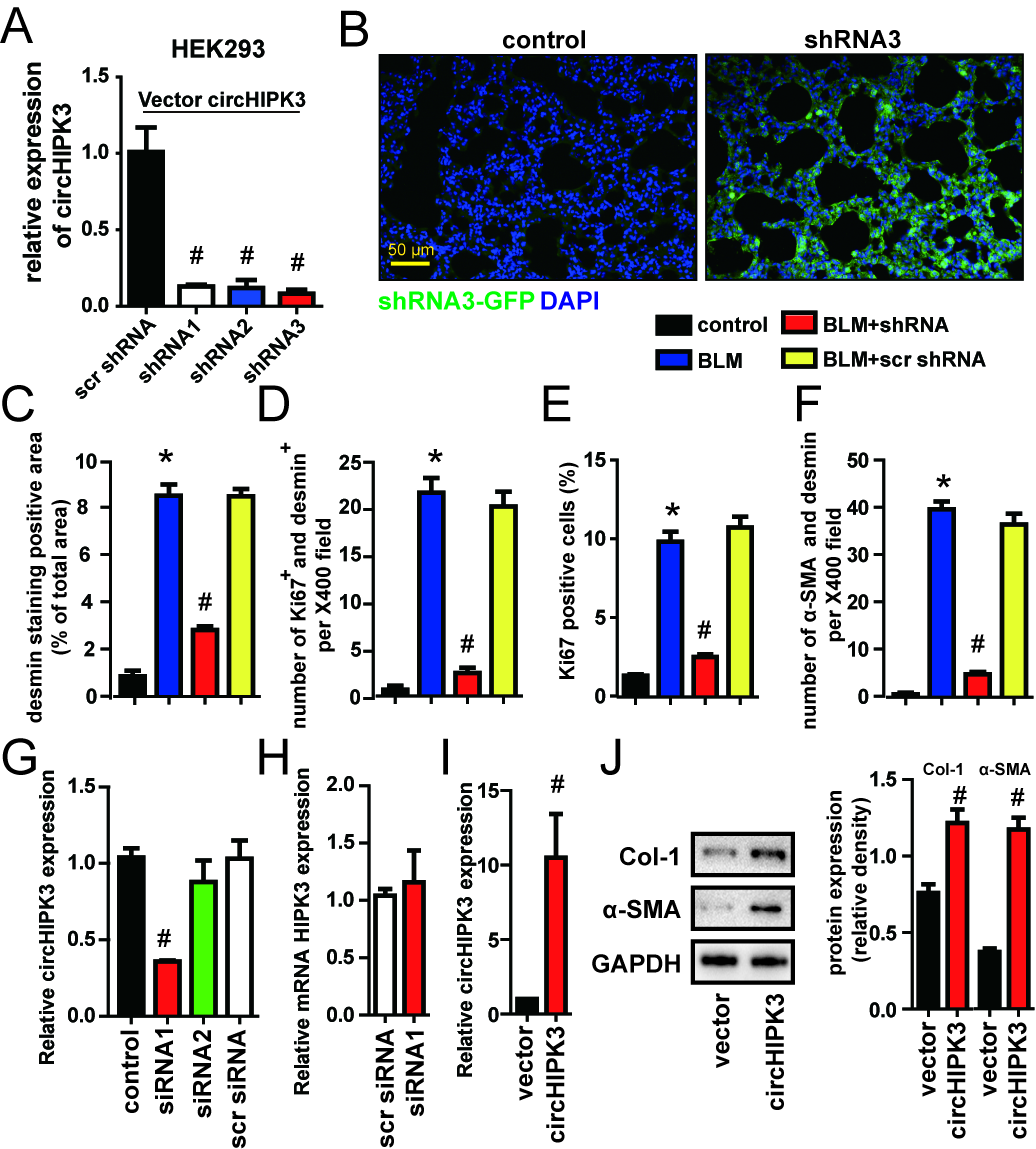

Supplement: Supplementary file 1 — Supplementary Figure 1 [file 41419_2019_1430_MOESM1_ESM.tif]

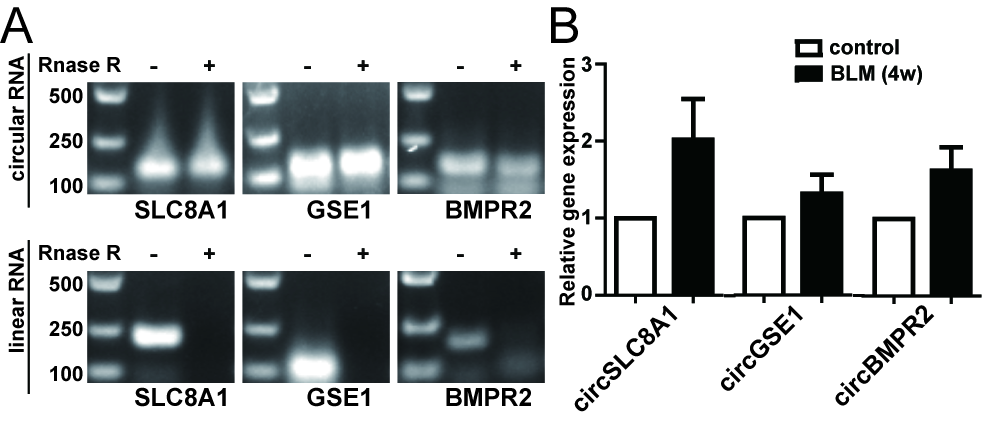

Supplement: Supplementary file 2 — Supplementary Figure 2 [file 41419_2019_1430_MOESM2_ESM.tif]
