# Supplementary material for: circHIPK3 regulates lung fibroblast-to-myofibroblast transition by functioning as a competing endogenous RNA
Source: Cell Death Dis. 2019 Feb 22;10(3):182. doi: 10.1038/s41419-019-1430-7 (PMC6385182; doi:10.1038/s41419-019-1430-7)
Supplement: Supplementary file 3 — supplemental figure legends [file 41419_2019_1430_MOESM3_ESM.docx]

**Supplementary Figure 1.**

(A) HEK-293T cells were co-transfected with pADM-circHIPK3 plus scrambled shRNA, circHIPK3 shRNA1, circHIPK3 shRNA2, circHIPK3 shRNA3. Silencing efficiency was dected by qRT-PCR (n=3, #p<0.05 versus scr shRNA). (B) 5-week old male mice were received an intratracheal injection of AAV6 containing circHIPK3 shRNA3-GFP, or scrambled shRNA. 3 weeks later, mice lung sections were viewed under a fluorescent microscope to assess the transduction efficiency.  (C-F) Data from Fig 3, G and H was quantified (n=4, *p<0.05 versus control, #p<0.05 versus BLM+scr shRNA). (G) Silencing efficiency of circHIPK3 siRNA1, siRNA2, scrambled siRNA were detected by qRT-PCR. (n=3, #p<0.05 versus scr siRNA). (H) WI-38 were transfected with circHIPK3 siRNA1 and scrambled siRNA. qRT-PCR was performed to detect the expression of HIPK3. (n=3, #p<0.05 versus scr siRNA). (I) WI-38 cells were transfected with pADM-GFP (vector) or pADM-circHIPK3. qRT-PCR was performed to detect the expression of circHIPK3. (n=3, #p<0.05 versus vector). (J) Myofibroblast markers, Col-1 and α-SMA were determined by western bolt. GAPDH was detected as the internal control. A representative immunoblot was shown (n=3, #p<0.05 versus vector). Data (C-F) are represented as means ± SD. Data (A, G-J) are represented as means ± SEM.

**Supplementary Figure 2.**

(A) Total RNAs isolated from normal mouse lung were digested by RNase R followed by RT-PCR detection. (B) qRT-PCR was performed to detect the expression of circSLC8A1, circGSE1 and circBMPR2 in the lung of normal and bleomycin-treated mice (n=4).
