# Supplementary material for: circHIPK3 regulates lung fibroblast-to-myofibroblast transition by functioning as a competing endogenous RNA
Source: Cell Death Dis. 2019 Feb 22;10(3):182. doi: 10.1038/s41419-019-1430-7 (PMC6385182; doi:10.1038/s41419-019-1430-7)
Supplement: Supplementary file 4 — Supplementary Table 1 [file 41419_2019_1430_MOESM4_ESM.doc]

Supplementary Table 1

| **Primer squence** |
| --- |
| circHIPK3 for mouse Forward 5'-GGATCGGCCAGTCATGTATC-3'  Reverse 5'-ACCGCTTGGCTCTACTTTGA-3'  HIPK3 mRNA for mouse Forward 5'-GTGATCCGGCCTGTTCTTCA-3'  Reverse 5'-TGACTGGCCGATCCAAAGTC-3'  GAPDH mRNA for mouse Forward 5'-GTCAAGGCTGAGAACGGGAA-3'  Reverse 5'-AAATGAGCCCCAGCCTTCTC-3'  circHIPK3 for human Forward 5'-TATGTTGGTGGATCCTGTTCGGC-3'  Reverse 5'-TGGTGGGTAGACCAAGACTTGTGA-3'  HIPK3 mRNA for human Forward 5'-GACCTGAGGAGATCAAGCCG-3'  Reverse 5'-ATTGGGGCCCATTCCTGAC-3'  GAPDH mRNA for human Forward 5'-CCAAGGTCATCCATGACAAC-3'  Reverse 5'-GCTTCACCACCTTCTTGATG-3'  SOX4 mRNA for mouse Forward 5'-CCTCAAGCACATGGCTGACTACC-3'  Reverse 5'-CACTGGACGGCGAGGAGGAG-3'  collagen1α1 mRNA for mouse Forward 5'-ACAATGCCGAGAACACGGAAGC-3'  Reverse 5'-TGAGGTGGCTGAGGCAGGAAG-3'  SOX4 mRNA for human Forward 5'-ACAATGCCGAGAACACGGAAGC-3'  Reverse 5'-GATCTGCGACCACACCATGAAGG-3'  collagen1α1 mRNA for human Forward 5'-ACAATGCCGAGAACACGGAAGC-3'  Reverse 5'-GATCTGCGACCACACCATGAAGG-3' |
| **siRNA squence** |
| circHIPK3 siRNA1 sense 5'-GGUACUACAGGUAUGGCCUTT-3'  antisense 5'-AGGCCAUACCUGUAGUACCTT-3'  circHIPK3 siRNA2 sense 5'-CUACAGGUAUGGCCUCACATT-3'  antisense 5'-UGUGAGGCCAUACCUGUAGTT-3'  Ago2 siRNA1 sense 5'-GACGGCAGGAAGAAUCUAUTT-3'  antisense 5'-AUAGAUUCUUCCUGCCGUCTT-3'  Ago2 siRNA2 sense 5'-GGGUAAAGUUUACCAAAGATT-3'  antisense 5'-UCUUUGGUAAACUUUACCCTT-3'  Ago2 siRNA3 sense 5'-GCCUGUAUCAAGCUAGAAATT-3'  antisense 5'-UUUCUAGCUUGAUACAGGCTT-3' |
| **shRNA** **target squence** |
| 5'-GATCCGCTACTACAGGTATGGCCTCACAATTCAAGAGATTGTGAGGCCATACCTGTAGTAGTTTTTTA-3' |
| **FISH probe** |
| circHIPK3 for human 5'-TGTGAGGCCATACCTGTACCGAGATTG-3' |
